# *Mycobacterium tuberculosis* epidemiology in Oman: whole-genome sequencing uncovers transmission pathways

**DOI:** 10.1128/spectrum.02420-23

**Published:** 2023-09-28

**Authors:** Hamza A Babiker, Amina Al-Jardani, Saleh Al-Azri, Robert A. Petit, Eltaib Saad, Sarah Al-Mahrouqi, Reham A.H. Mohamed, Salama Al-Hamidhi, Abdullah A. Balkhair, Najma Al Kharusi, Laila Al Balushi, Samiya Al Zadjali, Lucía Pérez-Pardal, Albano Beja-Pereira, Ahmed Babiker

**Affiliations:** 1 Biochemistry Department, College of Medicine and Health Sciences, Sultan Qaboos University, Muscat, Oman; 2 Institute of Immunology and Infection Research, School of Biological Sciences, University of Edinburgh, Edinburgh, United Kingdom; 3 Central Public Health Laboratories, National Tuberculosis Reference Laboratory, Directorate General for Disease Surveillance and Control, Ministry of Health, Muscat, Oman; 4 Wyoming Department of Health, Wyoming Public Health Laboratory, Cheyenne, Wyoming, USA; 5 Department of Medicine, Ascension Saint Francis Hospital, Evanston, Illinois, USA; 6 Department of Medicine, College of Medicine and Health Sciences, Sultan Qaboos University, Muscat, Oman; 7 CIBIO, Centro de Investigação em Biodiversidade e Recursos Genéticos, InBIO Labora­ tório Associado, Campus de Vairão, Universidade do Porto, Vairão, Portugal, Vairão, Portugal; 8 BIOPOLIS Program in Genomics, Biodiversity and Land Planning, CIBIO, Campus de Vairão, Vairão, Portugal; 9 DGAOT, Faculty of Sciences, Universidade do Porto, Porto, Portugal; 10 Division of Infectious Diseases, Department of Medicine, Emory University School of Medicine, Atlanta, Georgia, USA; 11 Department of Pathology and Laboratory Medicine, Emory University School of Medicine, Atlanta, Georgia, USA; Montefiore Medical Center and Albert Einstein College of Medicine, Bronx, New York, USA

**Keywords:** *Mycobacterium tuberculosis*, molecular phylogeography, phylogeny, Oman

## Abstract

Tuberculosis (TB) originating from expatriates that hail from high TB-burden countries is hypothesized to play a role in continued TB transmission in Oman. Here, we used whole-genome sequencing (WGS) to assess national TB transmission dynamics. The annual incidence per 100,000 population per year was calculated for nationals and expatriates. A convenience sample of *Mycobacterium tuberculosis* (MTB) isolates from 2018 to 2019 was sequenced and analyzed with publicly available TB sequences from Bangladesh, Tanzania, the Philippines, India, and Pakistan. Relatedness was assessed by generating core-genome single nucleotide polymorphism (SNP) distances. The incidence of TB was five cases per 100,000 persons in 2018 and seven cases per 100,000 persons in 2020 (*R^2^* = 0.34, *P* = 0.60). Incidence among nationals was 3.9 per 100,000 persons in 2018 and 3.5 per 100,000 persons in 2020 (*R^2^* = 0.20*, P* = 0.70), and incidence among expatriates was 7.2 per 100,000 persons in 2018 and 12.7 per 100,000 persons in 2020 (*R^2^* = 0.74, *P* = 0.34). Sixty-eight local MTB isolates were sequenced and analyzed with 393 global isolates. Isolates belonged to nine distinct spoligotypes. Two isolates, originating from an expatriate and an Omani national, were grouped into a WGS-based cluster (SNP distance < 12), which was corroborated by an epidemiological investigation. Relatedness of local and global isolates (SNP distance < 100) was also seen. The relatedness between MTB strains in Oman and those in expatriate countries of origin can aid inform TB control policy. Our results provide evidence that WGS can complement epidemiological analysis to achieve the End TB strategy goal in Oman.

**IMPORTANCE:**

Tuberculosis (TB) incidence in Oman remains above national program control targets. TB transmission originating from expatriates from high TB-burden countries has been hypothesized to play a role. We used whole-genome sequencing (WGS) to assess TB transmission dynamics between expatriates and Omani nationals to inform TB control efforts. Available *Mycobacterium tuberculosis* isolates from 2018 to 2019 underwent WGS and analysis with publicly available TB sequences from Bangladesh, the Philippines, India, and Pakistan to assess for genetic relatedness. Our analysis revealed evidence of previously unrecognized transmission between an expatriate and an Omani national, which was corroborated by epidemiological investigation. Analysis of local and global isolates revealed evidence of distant relatedness between local and global isolates. Our results provide evidence that WGS can complement classic public health surveillance to inform targeted interventions to achieve the End TB strategy goal in Oman.

## INTRODUCTION

Oman is a low-incidence tuberculosis (TB) country, where successful control measures have led to a substantial reduction in the disease burden. Nonetheless, since 2015, TB rates have lingered between 5.9 and 8.9 per 100,000 population (1), persisting above the national program control target of <1 per 100,000 population by 2035 (2). Around 400 cases are reported every year, with a larger proportion seen among expatriates (3), who represent over 40% of the population. The majority of expatriates in Oman originate from high TB-burden countries with a TB incidence greater than 100 cases per 100,000 population (4).

The TB epidemiology in Oman parallels the pattern seen in other Gulf Cooperation Council (GCC) countries (5 – 7). This has partly been attributed to the similar demographic structure and the large proportion of expatriates from high TB-burden countries (8). Expatriates constitute a vast proportion of GCC countries’ population reaching up to 37%, 46%, 53%, 86%, and >89% in Saudi Arabia, Oman, Bahrain, Qatar, and UAE, respectively (9). In 1997, the Gulf Health Council endorsed the goal of TB elimination, aiming at reducing the incidence of new cases among nationals to <1 per 100,000 by 2010. However, no country in the region has achieved the desired targets (1).

Molecular analysis of *Mycobacterium tuberculosis* (MTB) within the GCC has shown a high degree of relatedness of *M. tuberculosis* among nationals and expatriates. Studies in Saudi Arabia, Oman, and Kuwait revealed a high proportion of multinational genotype-matched clusters of *M. tuberculosis* lineages suggesting marked transmission permeability (6, 10, 11). Within Oman, a previous analysis of 1,295 MTB isolates revealed significant spoligoprofile clustering of isolates obtained from nationals and expatriates. Such clustering of isolates is suggestive of transmission events (11).

Whole-genome sequencing (WGS) can allow for higher resolution and more accurate calling of transmission events compared to standard genotyping (such as Mycobacterial Interspersed Repetitive Units Variable Number Tandem Repeats [MIRU-VNTR] typing), which has previously been shown to overestimate the rate of transmission (12). Here, we aimed to extend prior findings (11) and use WGS to assess for undetected transmission events to aid in TB control efforts.

## MATERIALS AND METHODS

### TB epidemiology in Oman

TB is a notifiable disease in Oman and all cases across the country are reported to the National TB program at the Directorate General for Disease Surveillance and Control Program at the Ministry of Health. All positive samples from the healthcare institutions are submitted to the National Reference TB laboratories at the Central Public Health Laboratories for the confirmation of identification by acid fast bacilli smear microscopy, mycobacterial culture and GeneXpert (Xpert MTB/RIF) assay, and drug susceptibility testing. TB cases per year and population estimates were obtained from the annual report of the Ministry of Health (3).

### *Mycobacterium tuberculosis* phenotypic and genomic characterization

A convenience sample of available MTB isolates from 2018 and 2019 were obtained from the National Tuberculosis Reference Laboratory at the Central Public Health Laboratories of the Ministry of Health in Oman and underwent phenotypic and genotypic characterization.

The laboratory performs confirmation of TB diagnosis using standard microbiological procedures for the identification of the *M. tuberculosis* complex and *in vitro* drug-susceptibility test for all first-line anti-TB drugs (13). Isolates resistant to a single drug were classified as mono-resistant. Isolates resistant to more than one drug but not to both isoniazid (INH) and rifampin (RIF) were classified as poly-resistant (PolyR). Isolates resistant to at least INH plus RIF were classified as multi-drug resistant (MDR).

### Whole-genome sequencing

Library preparation, sequencing, and analysis were performed by Novogene (UK) Company Limited. Library preparation was performed using the NEBNext DNA Library Prep Kit (New England BioLabs, USA). Index codes were added to each sample. The genomic DNA is randomly fragmented to a size of 350 bp. DNA fragments were end polished, A-tailed, ligated with adapters, size selected, and further PCR enriched. Then, PCR products were purified (AMPure XP system), followed by size distribution determination by Agilent 2100 Bioanalyzer (Agilent Technologies, CA, USA), and quantification using real-time PCR. The library was then sequenced on a NovaSeq 6000 S4 flow cell with PE150 strategy.

### Bioinformatics and phylogenetic analysis

Sequencing data were processed using the Bactopia pipeline (v1.2.2) (14). Briefly, input FASTQs underwent processing and removal of Illumina-related adapters and phiX contaminants using BBTools (15). Contigs were then assembled by the Shovill pipeline (v1.0.7) (16) using strategic k-mer extension for scrupulous assemblies (v2.3.0) (17). The assembly was then assessed for its biological (e.g., containment and contamination) as well as its technical (e.g., misassembles and errors) quality using CheckM (18) and QUAST (19). A summary of the sequence statistics and assembly statistics was computed and the rank of Gold, Silver, Bronze, or Fail was assigned based on sequence and assembly quality (Table S1). Annotation was performed by Prokka (20), supplemented by clustered protein annotations from completed *M. tuberculosis* genomes available from RefSeq (21). Variants were predicted using Snippy (22) and BWA (23) by alignment of the QC'd FASTQs to the downloaded reference genome (GCF_000195955.2). Bedtools (24) was used to generate the per-base coverage of the reference alignment, and vcf-annotator was used to add annotations to the final VCF (25). SAMtools (26) was then used to convert the alignments from SAM to BAM files. The presence of antimicrobial resistance gene, which predicts resistance to anti-tuberculosis drugs, and determination of lineage were performed using TBProfiler (27).

To examine the relatedness of *M. tuberculosis* lineages in Oman with those in the expatriate’s countries of origin, we created a database of publicly available isolates from Bangladesh, Tanzania, the Philippines, India, and Pakistan. We downloaded the raw FASTQs from the Sequence Read Archive (SRA) (28) (*n* = 593, Table S2) and processed them as above. Publicly available TB sequences were compared with local isolates and filtered out of downstream pangenome analysis if average nucleotide identity (ANI) was less than 95% as determined by FastANI (29) (Table S3). Phylogenetic trees were constructed based on a core gene alignment identified by Roary (30). Using IQ-Tree (31), a maximum likelihood tree was generated by running 1,000 bootstrap replicates under the generalized time-reversible model of evolution. The tree was visualized and annotated using Interactive Tree Of Life (32). Potential transmission between patients was assessed by generating a core-genome SNP distance matrix for local isolates (*n* = 68) calculated using snp-dists (33). An SNP distance of <12 was considered as a WGS cluster and potential case of transmission (12, 34).

A network analysis was performed using a few representative global strains from each lineage (Table S6). Set parsimony splits were computed (35). All positions containing gaps and missing data were eliminated. SplitsTree4 (version 4.15.1) (36) was used to generate a neighbor-net genetic network based on the alignment of positions using the uncorrected *p*-distance and 1,000 replicates (36).

### Statistical analysis

Descriptive analyses were performed to summarize specimen information, demographics, microbiological, and genotyping results. Statistical analysis was performed using R version 4.0.2 (Vienna, Austria) and the RStudio interface version 1.3.1073 (Boston, MA, USA)

## RESULTS

### TB in Oman, 2018–2020

**Fig 1:**
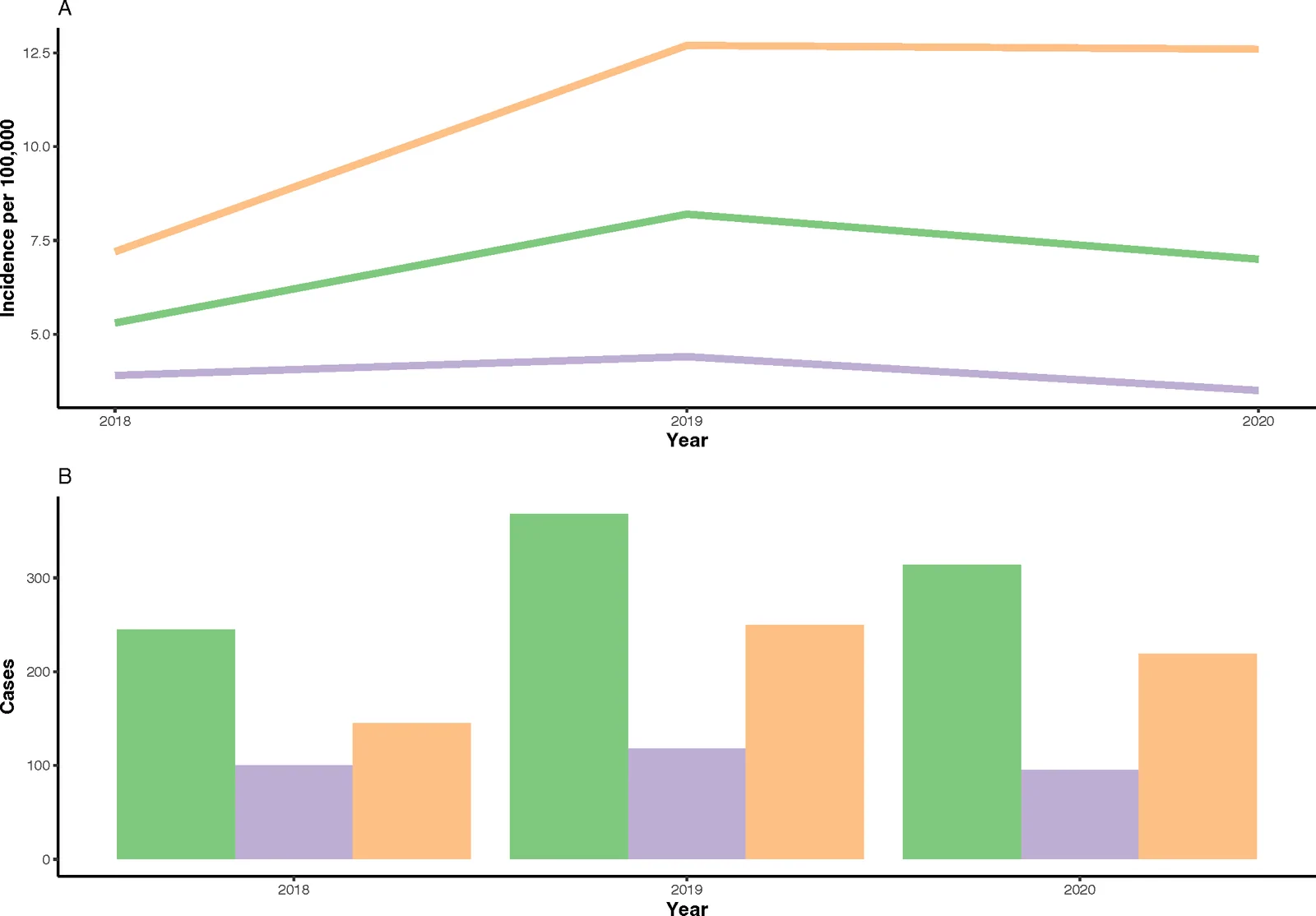
TB incidence per 100,000 population (**A**) and cases (**B**) in Oman between 2018 and 2020 for total population (green), nationals (purple), and expatriates (orange).

The incidence of TB in Oman was 12 cases per 100,000 in 2011 and reached its lowest level of 5.9 per 100,000 population in 2021. Between 2018 and 2020, the time period that mirrors isolate availability, there were 927 reported cases of tuberculosis in Oman, 313 (33%) among nationals and 614 (66%) among expatriates. The overall annual incidence of TB was five cases per 100,000 persons in 2018 and seven cases per 100,000 persons in 2020 (*R^2^* = 0.34, *P* = 0.60). The incidence of TB among nationals was 3.9 in 2018 and 3.5 per 100,000 persons in 2020 (*R^2^* = 0.20*, P* = 0.70). The incidence among expatriates was 7.2 per 100,000 persons in 2018 and 12.7 per 100,000 in 2020 (*R^2^* = 0.74, *P* = 0.34) (Fig. 1A and B).

### Clinical characteristics of MTB patients with available isolates

A total of 69 MTB isolates from unique patients with active TB were obtained for WGS. The MTB isolates were obtained from 11 governorates in Oman, from Omani nationals (*n* = 17, 25%) and expatriates (*n* = 52, 75%), collected between 2018 (*n* = 36, 52%) and 2019 (*n* = 33, 48%).

The patients were aged between 16 and 88 years. The median (interquartile range, IQR) age of nationals and expatriate patients was 33 (16) and 37 (37) years, respectively. The majority of the patients were male (*n* = 49, 72.0%) with a higher percentage of males among both groups (expatriates: 72.5%, Omani nationals: 82.3%). The majority of the patients had pulmonary TB (*n* = 59, 87%). Key clinical and epidemiological features of patients included in the analysis (*n* = 69) with available clinical/epidemiological data (*n* = 68) are summarized in Table 1.

**TABLE 1:** Clinical and microbiological characteristics of patients with sequenced TB isolates (*n* = 68)^*b*^

Variable	Total population *N* = 68^*a*^ (%)	Oman nationals *N* = 17 (25)	Expatriates *N* = 51 (75)
Median age (IQR)	35 (15)	33 (16)	37 (37)
Sex (male)	49 (72)	13 (76)	36 (71)
Year of collection			
2018	36 (53)	14 (82)	22 (43)
2019	32 (47)	3 (18)	28 (55)
Infection site			
Pulmonary	59 (87)	12 (71)	47 (71)
Extra-pulmonary	9 (13)	5 (29)	4 (8)
Nationality status			
Expatriates			
India	16 (31)	0 (0)	16 (31)
Bangladesh	14 (27)	0 (0)	14 (27)
Pakistan	6 (12)	0 (0)	6 (12)
Filipino	4 (8)	0 (0)	4 (8)
Indonesia	1 (2)	0 (0)	1 (2)
Brunei	1 (2)	0 (0)	1 (2)
Yemen	1 (2)	0 (0)	1 (2)
Egypt	1 (2)	0 (0)	1 (2)
Sudan	1 (2)	0 (0)	1 (2)
Uganda	1(2)	0 (0)	1 (2)
Tanzania	1 (2)	0 (0)	1 (2)
Omanis	17 (25)	17 (100)	0 (0)
Spoligotype			
Beijing	3 (4)	0 (0)	3 (6)
CAS	11 (16)	5 (29)	6 (12)
EAI	36 (52)	9 (53)	26 (49)
LAM;T;S;X;H	19 (28)	3 (18)	16 (31)
Drug susceptibility profile			
Sensitive	22 (32)	9 (53)	13 (25)
INHR	28 (41)	2 (12)	15 (29)
RIFR	4 (8)	0 (0)	1 (5.9)
MDR	3 (4)	0 (0)	3 (6)
PolyR	11 (16)	19 (20)	1 (6)

^*a*^ Clinical data not available for one associated isolate.

^*b*^ IQR: interquartile range, INHR: Isoniazid mono-resistant, MDR: multi-drug resistant, PolyR: polydrug resistant, and RIFR: Rifampin mono-resistant.

### Antimicrobial susceptibility profiles

The isolates had variable antimicrobial susceptibility profiles. Twenty-two isolates (32%) were pan-susceptible, 17 isolates (24%) were INH mono-resistant, one (1%) isolate was RIF mono-resistant, 11 isolates (16%) were poly-resistant, and three isolates (4%) were MDR (Table 1). Antimicrobial resistance genes and mutations associated with resistance that were detected are presented in Table 2.

**TABLE 2:** TB isolate genotypic characteristics

Characteristic	*N* (%)
Lineage	
Lineage1	36 (52)
Lineage2	3 (4)
Lineage3	11 (16)
Lineage4	19 (28)
Family	
East-African-Indian	11 (16)
East-Asian	3 (4)
Euro-American	19 (28)
Indo-Oceanic	36 (52)
Spoligotype	
Beijing	3 (4)
CAS	11 (16)
EAI	36 (52)
LAM;T;S;X;H	19 (28)
Rifampin resistance-associated mutations	
rpoB	4 (6)
Isoniazid resistance-associated mutations	
fabG1 c.-15C > T	13 (19%)
inhA p.Ile21Thr (1.00), ahpC p.Glu76Lys	1 (1)
katG	12 (17)
Ethambutol resistance-associated mutations	
embB	7 (10)
Pyrazinamide-resistance-associated mutations	
pncA	4 (6)
Streptomycin resistance-associated mutations	
gid c.	8 (11)
rpsL	10 (14)
rrs r.	2 (3)
Fluoroquinolone resistance-associated mutations	
gyrB p.Thr500Asn	1 (1)
Capreomycin resistance-associated mutations	
tlyA p.Leu118Pro	1 (1)
Ethionamide resistance-associated mutations	
fabG1 c.-15C > T	12 (17)
inhA p.Ile21Thr	1 (1)
Para-aminosalicylic acid_mutations	
folC p.Ile43Ser	1 (1)

### Spoligotypes of *M. tuberculosis*

**Fig 2:**
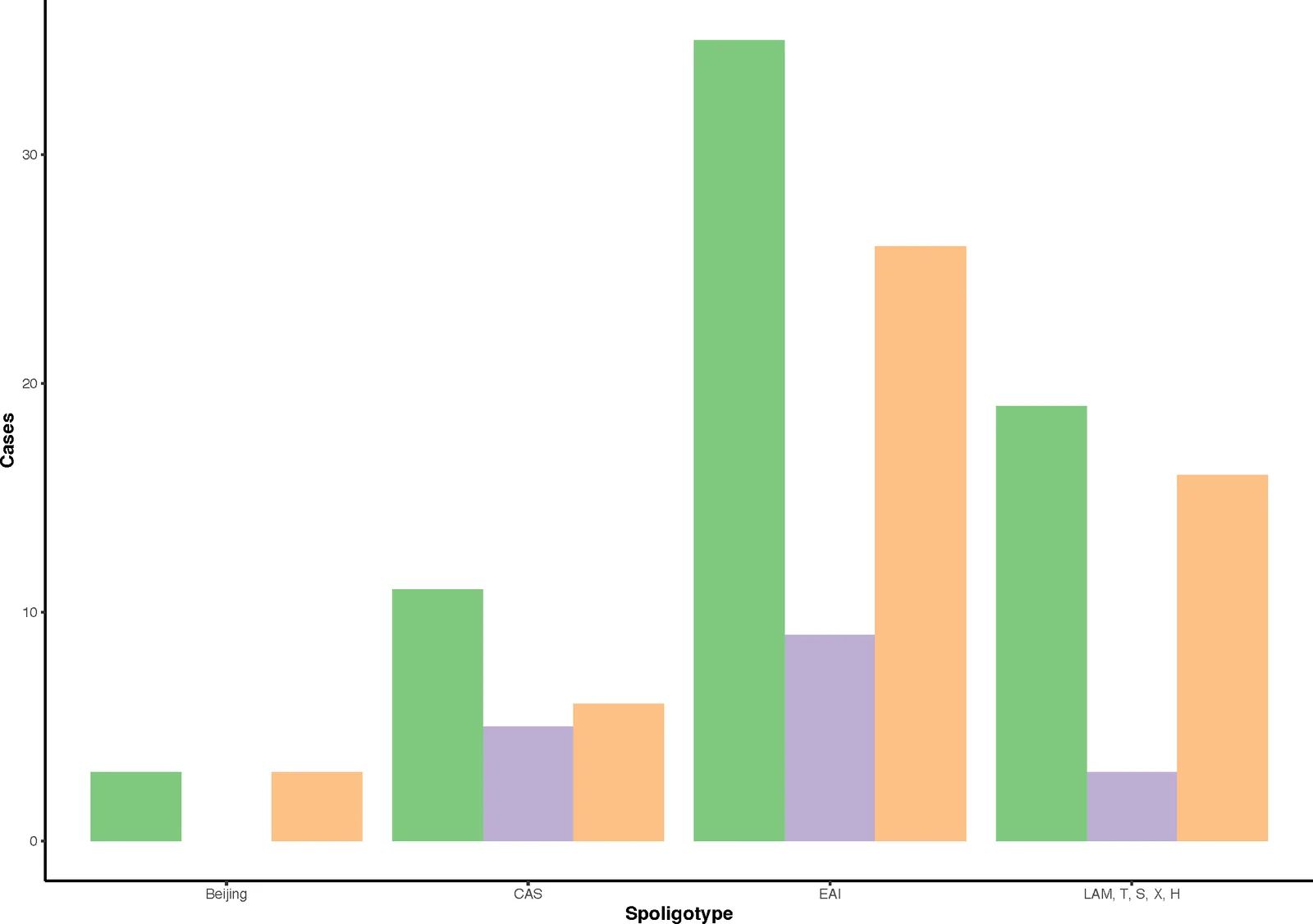
Distribution of *M. tuberculosis* spoligotypes for the total population (green), nationals (purple), and expatriates (orange).

*M. tuberculosis* isolates from Oman comprised four lineages: Indo-Oceanic (*n* = 35, 51%), Euro-American (*n* = 19, 28%), East Asian Indian (*n* = 11, 16%), and East-Asian (*n* = 3, 4%). These were further divided into nine sub-lineages (spoligotypes), the majority of *M. tuberculosis* belong to three clades; East African Indian (EAI) (*n* = 35, 51%), Central Asia Strain (CAS) (*n* = 11, 16%), and Beijing (*n* = 3, 4%). Other less common lineages identified included T family, Latin American Mediterranean (LAM), Haarlem (H), X family, S family, and MANU (Fig. 2; Table 2).

Two of the major sub-lineages (CAS and EAI), as well as five sub-lineages (LAM, Manu, H, S, and X1), were shared between Omani nationals and expatriates (Fig. 2). The Beijing sub-lineage was exclusive to expatriates. At least seven (CAS, EAI, LAM, Manu, H, S, and X1) of the nine clades detected contained isolates from both Omani nationals and expatriates.

### Relatedness of MTB isolates in Oman

Of the 69 isolates sequenced, one isolate was removed from downstream analysis due to poor assembly metrics, specifically too many contigs (*n* = 2,268) (Table S1).

**Fig 3:**
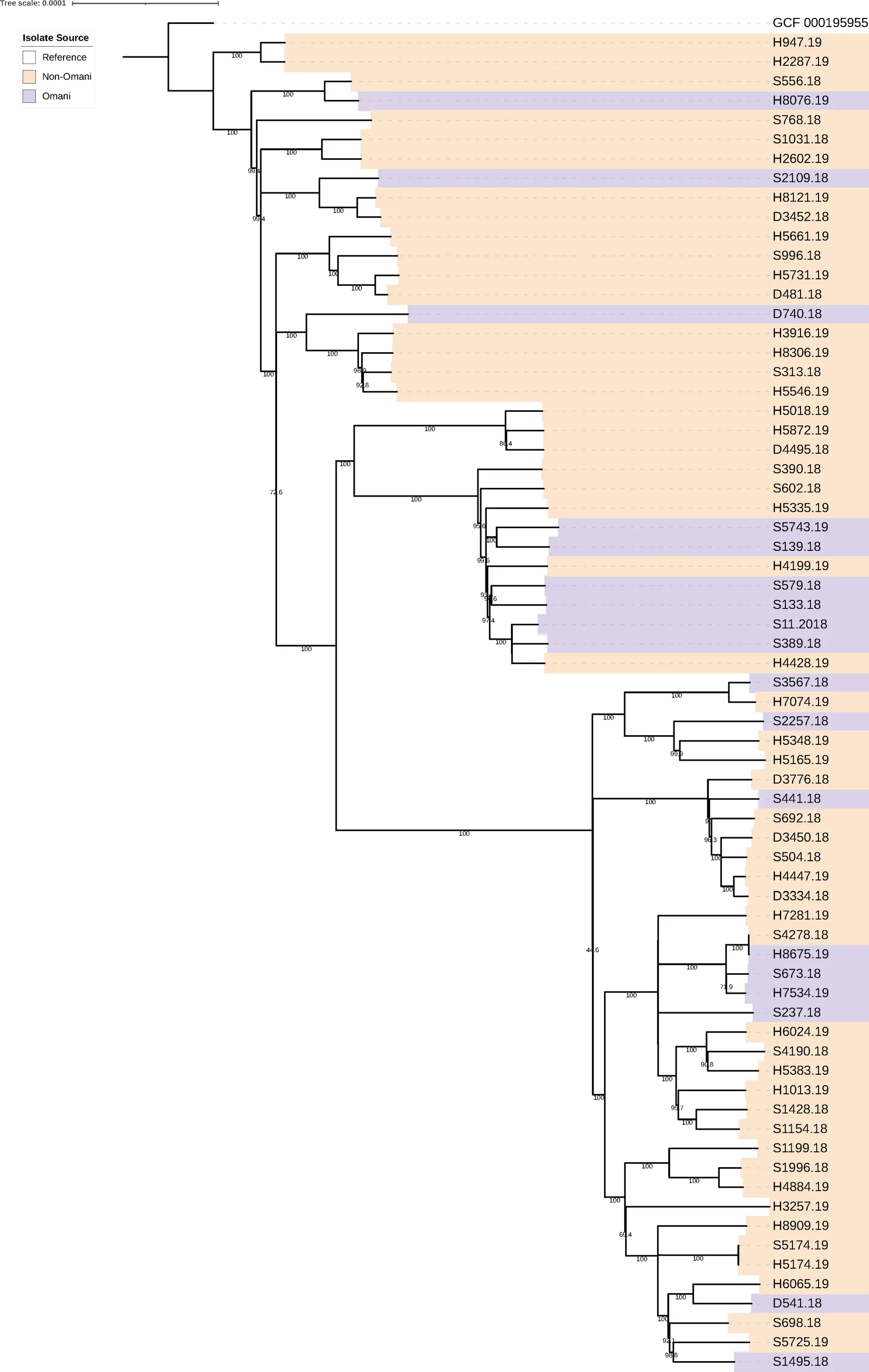
Phylogenetic tree of *M*. *tuberculosis* from Oman, 2018–2019 (*n* = 69). Phylogenetic trees were constructed based on a core genome alignment identified by Roary (30). Using IQ-Tree (31), a maximum likelihood tree was generated by running 1,000 bootstrap replicates under the generalized time-reversible model of evolution. The tree was visualized and annotated using Interactive Tree of Life (32). The tree is annotated based on isolate source (white: reference, purple: Omani nationals, and orange: expatriates).

The core-genome SNP-wise comparison of 66 out of the remaining 68 isolates demonstrated a difference of more than 100 SNPs with every other isolate, thus, reducing the high spoligo-based estimates of transmission by 100%. Phylogenetic analysis and SNP difference revealed an instance of possible transmission due to the low number of SNP count (<12) and clustering on the phylogenetic tree between two remaining isolates (Table S4; Fig. 3). The isolates were from a female Omani national (sample H8675_19) and a female expatriate (sample S4278), both from the Muscat region with a similar AST phenotype (INHR) and had one SNP difference. The epidemiological investigation revealed key data, including residence location and behaviors, which suggested a previously unknown transmission link between these two cases.

### *M. tuberculosis* in Oman and other regional strains

We examined the relatedness between *MTB* isolates in Oman and those in the countries of origin of expatriates. A total of 593 available *M. tuberculosis* sequences from Bangladesh, Tanzania, the Philippines, India, and Pakistan were downloaded from SRA and processed using the Bactopia pipeline. After filtering for low quality genomes and those that did not meet ANI cutoff >95% similarity with local TB isolates, we were left with 393 isolates (Table S5).

**Fig 4:**
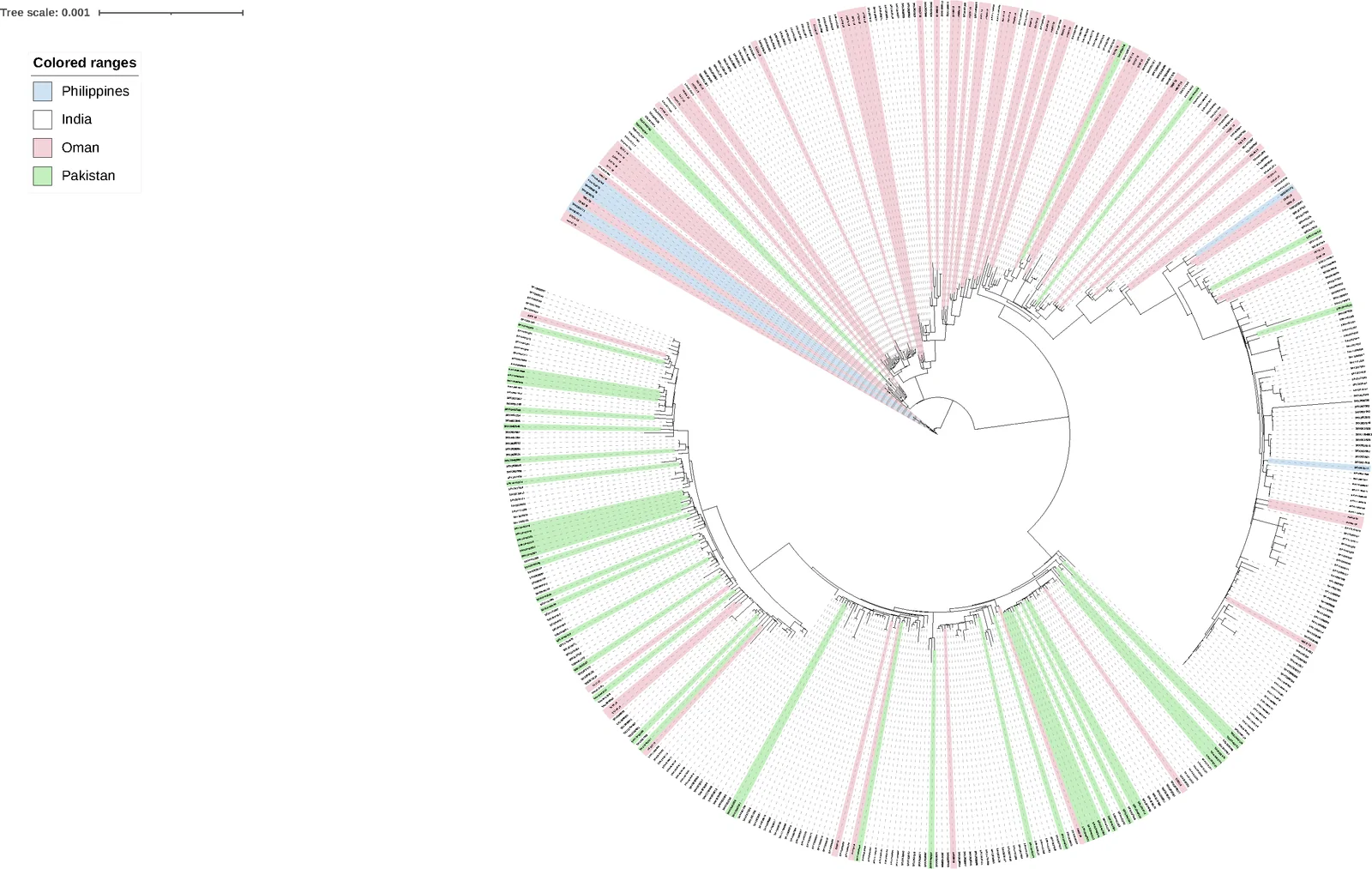
Phylogenetic tree of *M. tuberculosis* from Oman and globally available isolates from India, Pakistan, and the Philippines (*n* = 462). Phylogenetic trees were constructed based on a core genome alignment identified by Roary (30). Using IQ-Tree (31), a maximum likelihood tree was generated by running 1,000 bootstrap replicates under the generalized time-reversible model of evolution. The tree was visualized and annotated using Interactive Tree of Life (32). The tree is annotated based on country isolate source (blue: India, purple: the Philippines, green: Pakistan, and red: Oman).

**Fig 5:**
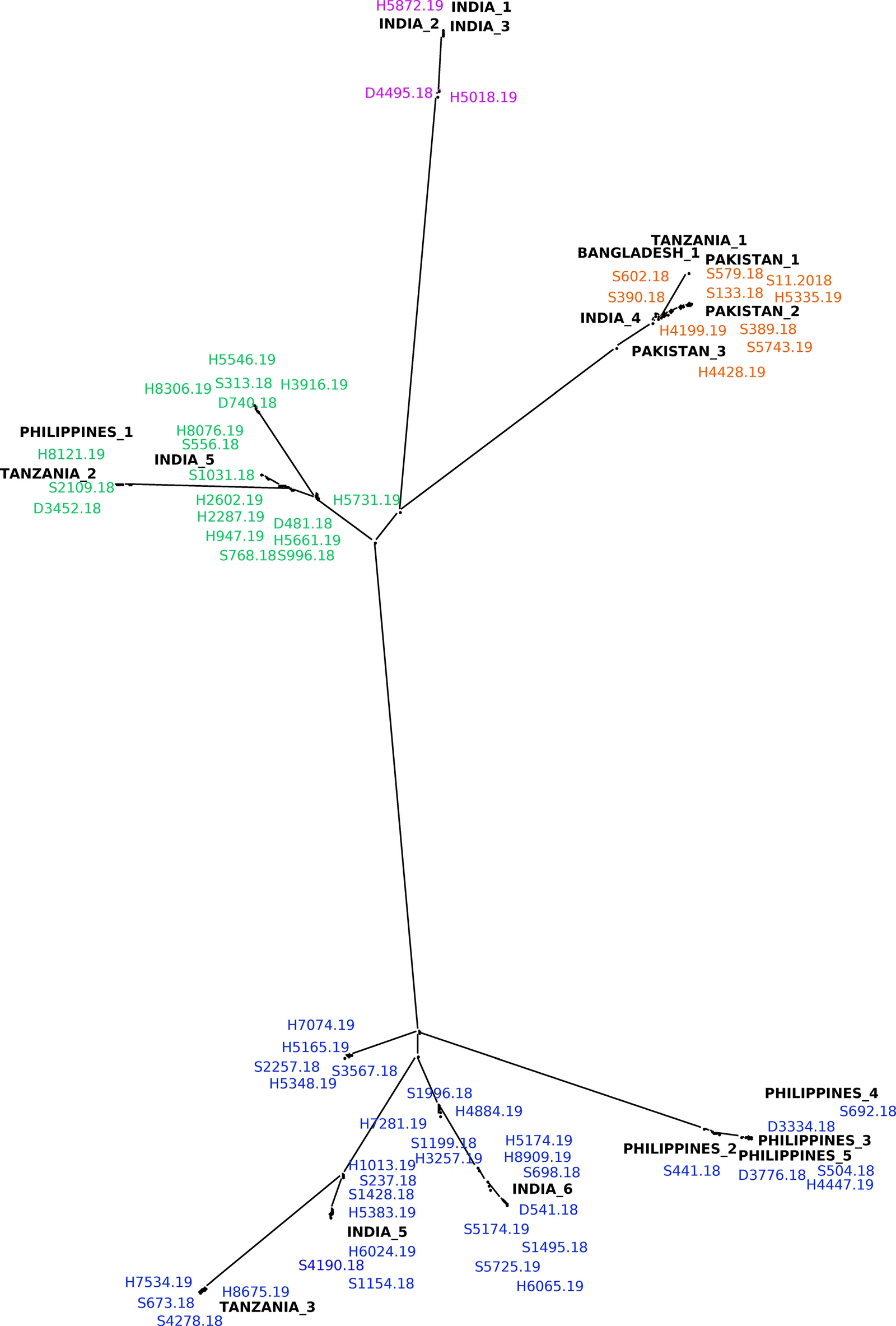
Neighbor-net genetic network of *M. tuberculosis* from Oman and down-sampled publicly global available isolates from India, Pakistan, Tanzania, and the Philippines. All positions containing gaps and missing data were eliminated. SplitsTree4 (version 4.15.1) (36) was used to generate a neighbor-net genetic network based on the alignment of 111 positions using the uncorrected *p*-distance and 1,000 replicates. The analysis involved 69 nucleotide sequences. The colors of the isolated are given according to their assignment to main TB lineages. Blue for lineage 1, violet for lineage 2, orange for lineage 3, and green for lineage 4. Names in bold black letters represent the country of origin of down-sampled publicly global available isolates (Table S6).

Phylogenetic analysis revealed no evidence of direct transmission. Clustering on the phylogenetic tree and an SNP distance of <100 with global genomes were seen in a few of the expatriate’s cases. Isolate H5872_19 (expatriate from Sharqiyah region, Oman) clustered with many isolates from India and had an SNP distance of <100. Isolate S390_18 (expatriate from Dhofar region, Oman) had an SNP distance of <100 with a genome from India (accession SRX2638073). Sample D3450_18 (expatriate) had an SNP distance of <100 SNPs with a genome from the Philippines (accession SRX2635773) (Table 2; Fig. 4), suggesting remote relatedness between these cases, which was supported by network analysis (Fig. 5).

## DISCUSSION

The low and persistent TB incidence profile of Oman has been attributed to the transmission of *M. tuberculosis* strains from the large proportion of expatriates from high TB-burden countries (37). Using WGS, the present study provides direct proof of a transmission event between an expatriate and an Omani national and revealed a degree of relatedness between MTB strains in Oman isolated from expatriates and those in expatriate’s home countries.

The majority of TB cases reported in Oman in 2019 and 2020 were seen among expatriates (1). In Oman, control of imported *M. tuberculosis* strains via expatriates is regulated by mandatory health checkups for initial visa approvals as well as renewal of residence visas (38, 39). However, latent TB (LTB) testing is not part of the current policy although it was seen as an important measure to reduce the high incidence of TB among expatriates (37). Testing for interferon-gamma release assay among 1,042 applicants for visa renewal in 2019, in Sohar governorate, showed a high prevalence of positive cases among expatriates from Asia (21%) and Africa (31%), indicative of LTB (38). Similarly, a survey of 501,290 expatriates, for visa renewal in 2018–2020, in Muscat governorate, revealed an LTB rate of 10.6 per 100,000 applicants (4). This is consistent with the findings of 158 out of 97,100 X-ray results of expatriates in Oman, with presumptive pulmonary TB, in 2018 (38). Thus, the diagnosed TB cases in Oman among the expatriate community are most probably a result of the reactivation of LTB acquired in their country of origin (4, 38).

Many of the isolates were found to belong to *M. tuberculosis* lineages, including EAI (51.5%), CAS (16.1%), and Beijing (4.4%), which are common in the country of origin of expatriates in Oman, such as the Indian subcontinent, South East Asia, and East Africa (40). Whereas the other lineages, T, LAM, and H (27.9%) are prevalent in East Africa, which has a historical link with Oman (41). These findings are consistent with a recent analysis of 1,295 *M. tuberculosis* isolates in different provinces in Oman, where no differences were seen among MTB lineages infecting nationals and expatriates (13). Such a pattern has also been reported in other GCC countries, with a similar demographic structure of a high proportion of expatriates, such as Saudi Arabia and Kuwait (6, 42). This contrasts reports from Europe and US, where MTB lineage isolates from expatriate patients were rarely found among those of local patients (43, 44). Thus, the high similarity of MTB lineages infecting expatriates and nationals in GCC countries maybe indicative of closer social interactions between nationals and expatriates. This is evident by the close genetic relatedness between some of the MTB lineages in Oman and other countries, with differences of <100 SNPs (Table 3) and clustering together on network analysis.

**TABLE 3:** *M. tuberculosis* in Oman and other global or regional isolates with an SNP distance of <100 SNPs^*a*^

Local *M. tuberculosis* isolates (Omani or expatriate)	Global/regional *M. tuberculosis* isolates (isolate source)	SNP distance
D3450 (expatriate)	SRX2635773 (the Philippines)	75
S390 (expatriate)	SRX2638073 (India)	92
H5872 (expatriate)	SRX11666884 (India)	82
	SRX11666892 (India)	82
	SRX11666895 (India)	79
	SRX11764878 (India)	85
	SRX11764881 (India)	93
	SRX11806095 (India)	66
	SRX11806096 (India)	87
	SRX11806098 (India)	94
	SRX11806100 (India)	85
	SRX11806101 (India)	79
	SRX11806108 (India)	86
	SRX11806109 (India)	91
	SRX11806110 (India)	92
	SRX11806114 (India)	83
	SRX11806116 (India)	93

^*a*^ SNP: single nucleotide polymorphism.

It is, thus, intuitive to hypothesize that frequent TB transmission between nationals and expatriates is occurring, given the higher number of genotype-matched clusters with shared MTB lineages of the two groups [7 out of 9 (77.7%)] (Fig. 2). However, spoligotyping can overestimate recent transmission due to its low discriminatory power. A recent analysis of 192 *M. tuberculosis* isolates in 41 spoligo-clusters, by MIRU-VNTRs genotyping, revealed no evidence of identical genotypes (11). Using the more sensitive WGS, the present study detected a small cluster of transmission (2 out of 68), thus reducing the probability of estimate of transmission to 2.9%. Our results are likely biased by sampling methodology and the use of a convenience sample (vs the ability to sequence all available isolates) and may underestimate transmission. Our findings are in line with the recent data from Oman (45) but build on prior efforts by inclusion of global sequences and providing granular SNP distances to define transmission (45).

In conclusion, the use of a more discriminatory typing approach by WGS can complement epidemiological methods to investigate transmission dynamics and identify clusters of recent transmission, for targeted interventions. In addition, WGS can provide data distinguishing between the reactivation of LTB from the country of origin and a recently acquired infection locally (46). This is particularly relevant to the End TB strategy in Oman, which aims at a 10% reduction per year, among nationals and expatriates, to achieve the target of <1 per 100,000 in 2035 (2).

## Data Availability

All locally generated sequences were deposited in SRA under BioProject accession PRJNA977967.

## SUPPLEMENTAL MATERIAL

Supplemental legends spectrum.02420-23-s0001.docx Legends for Tables S1 to S6.

Table S1 spectrum.02420-23-s0002.docx Assembly and annotation quality characteristics of sequenced TB isolates.

Table S2 spectrum.02420-23-s0003.xlsx Accession numbers of isolates downloaded from SRA.

Table S3 spectrum.02420-23-s0004.xlsx Final list of publicly available and local genomes used in pangenome analysis.

Table S4 spectrum.02420-23-s0005.xlsx Core-genome SNP distance matrix for local isolates.

Table S5 spectrum.02420-23-s0006.xlsx Core-genome SNP distance matrix for local and global isolates.

Table S6 spectrum.02420-23-s0007.xlsx Accession numbers of publicly available isolates used in network analysis.

ASM does not own the copyrights to Supplemental Material that may be linked to, or accessed through, an article. The authors have granted ASM a non-exclusive, world-wide license to publish the Supplemental Material files. Please contact the corresponding author directly for reuse.
