# Supplementary material for: Mycobacterium tuberculosis epidemiology in Oman: whole-genome sequencing uncovers transmission pathways
Source: Microbiol Spectr. 2023 Sep 28;11(5):e02420-23. doi: 10.1128/spectrum.02420-23 (PMC10581073; doi:10.1128/spectrum.02420-23)
Supplement: Supplemental legends — Legends for Tables S1 to S6. [file spectrum.02420-23-s0001.docx]

**Supplementary Tables Titles and Legends**

**Supplementary Table 1: Assembly and annotation quality characteristics of sequenced TB isolates (n=70)**

Sequencing data was processed using the Bactopia pipeline. The assembly was then assessed for its biological (e.g. containment & contamination) as well as its technical (e.g. misassemblies and errors) quality using CheckM and QUAST. A summary of the sequence statistics and assembly statistics were computed and rank of Gold, Silver, Bronze, or Fail was assigned based on sequence and assembly quality

**Supplementary Table 2: Accession numbers of Isolates downloaded from SRA (n=593).**

To examine the relatedness of M. tuberculosis lineages in Oman with those in the expatriate’s countries of origin, we created a database of publicly available isolates from Bangladesh, Tanzania, Philippines, India and Pakistan. We downloaded the raw FASTQs from the Sequence Read Archive

**Supplementary Table 3: Final list of publicly available and local genomes used in pangenome analysis**

Downloaded publicly available TB sequences were compared with local isolates and filtered out of downstream pangenome analysis if average nucleotide identity (ANI) was less than 95% as determined by FastANI

**Supplementary Table 4 Title: Core-genome SNP distance matrix for local isolates (n=68).**

Potential transmission and relatedness were assessed by generating a core-genome SNP distance matrix calculated using snp-dists. A snp distance < 12 was considered as a WGS cluster and potential case of transmission.

**Supplementary Table 5: Core-genome SNP distance matrix for local and global isolates (n=462)**

Potential transmission and relatedness between were assessed by generating a core-genome SNP distance matrix calculated using snp-dists. A snp distance < 12 was considered as a WGS cluster and potential case of transmission.

**Supplementary Table 6: Accession numbers of publicly available isolates used in network analysis (n=28).**

A network analysis was preformed using a few representative global strains from each lineage. Set parsimony splits were computed. All positions containing gaps and missing data were eliminated. SplitsTree4 was used to generate a neighbour-net genetic network based on the alignment of positions using the uncorrected p-distance and 1,000 replicates
