# Supplementary material for: Mycobacterium tuberculosis epidemiology in Oman: whole-genome sequencing uncovers transmission pathways
Source: Microbiol Spectr. 2023 Sep 28;11(5):e02420-23. doi: 10.1128/spectrum.02420-23 (PMC10581073; doi:10.1128/spectrum.02420-23)
Supplement: Table S1 — Assembly and annotation quality characteristics of sequenced TB isolates. [file spectrum.02420-23-s0002.docx]

**Supplementary Table 1: Assembly and Annotation Quality Characteristics of Sequenced TB isolates (n=70)**

| **Variable** | **Median(Q1,Q3)/ N(%)** |
| --- | --- |
| Total base pairs | 441,139,240 (441,139,155, 441,139,308) |
| Number of contigs (>1k) | 96 (88, 103) |
| N50 | 97,229 (83,175, 102,257) |
| GC content (%) | 65.5 (65.5, 65.7) |
| Summary assembly scores |  |
| Gold | 15 (22) |
| Silver | 53 (77) |
| Bronze | 0 (0) |
| Exclude* | 1 (3) |

*One sample was removed from the downstream analysis due to a high number of contigs (n=2268)

**Supplementary Table 1 Legend:** Sequencing data was processed using the Bactopia pipeline. The assembly was then assessed for its biological (e.g. containment & contamination) as well as its technical (e.g. misassembles and errors) quality using CheckM and QUAST. A summary of the sequence statistics and assembly statistics were computed, and rank of Gold, Silver, Bronze, or Fail was assigned based on sequence and assembly quality.
